# Single-stage “Fix and Flap” gives Good Outcomes in Grade 3B/C Open Tibial Fractures: A Prospective Study

**DOI:** 10.5704/MOJ.2003.010

**Published:** 2020-03

**Authors:** J Singh, MS Dhillon, SS Dhatt

**Affiliations:** 1 Department of Orthopaedics, Vardhman Mahavir Medical College and Safdarjung Hospital, New Delhi, India; 2 Department of Orthopaedics, Post Graduate Institute of Medical Education and Research, Chandigarh, India

**Keywords:** open tibial fractures, single-stage, limb salvage, external fixation, free flaps

## Abstract

**Introduction:**

Grade 3B/C open tibial fractures with grossly contaminated degloving injuries have poor outcomes, with or without vascular injuries. Treatment decision oscillates between limb salvage and amputation. The standard protocol of repeated debridement and delayed wound cover is a challenge in developing countries due to overcrowded emergencies and limited operating room availability. We present results of our modified protocol involving primary stabilisation with external fixation and immediate wound cover as an aggressive modality of treatment.

**Material and Methods:**

Thirty-three patients with severe open tibial shaft fractures were managed using a standardised protocol of emergent debridement, external fixation and immediate wound cover with free distant/local rotational muscle flaps and fasciocutaneous flaps, and with vascular repair in Grade 3C fractures. Intra-articular fractures were excluded. Patients were followed for a minimum of three years, with an assessment of clinical, radiological and functional outcomes.

**Results:**

Wound cover was achieved with 24 distant free muscle flaps, four local rotational muscle flaps and five fasciocutaneous flaps. All fractures united with an average time to union of 40.3 weeks (16-88). Fifteen patients (45.4%) underwent only a single major surgery using primary definitive external fixation. Deep infection was seen in four patients (12.1%). Nineteen patients had excellent to good outcomes, six were fair, and eight were poor.

**Conclusion:**

“Fix and Flap” in the same sitting, using immediate wound cover and external fixation, has given good results in our hands despite the delayed presentation, the neurovascular deficit and the degloving injury. This may be a better management strategy in overcrowded tertiary care centres of developing countries, with a single surgical procedure in almost half the cases.

## Introduction

Grade 3 open fracture is a high-energy injury, which may threaten the limb and occasionally even the life of the patient^1, 2^. The tibia is the commonest site for open fractures^3^ with grade 3B/C injuries being complicated by degloving of soft tissues, with gross contamination, possible vascular injuries, and often with poor outcomes. The management decision oscillates between limb salvage and amputation^4^, where many authors^5-9^ project a dismal outcome due to the medium and long-term problems with soft-tissue cover, infection, and union, ending with serious disability. The situation is even more challenging in developing countries like India where a late presentation of patients, lack of adequate tertiary care facilities, complex fracture patterns associated with poly-trauma, poor hygiene, poly-microbial infected wounds and antibiotic resistance, determine the final outcome^10^. The standard protocols of repeated serial debridement and delayed wound cover^11^, present a challenge in the underdeveloped countries, with the overcrowding and long waiting lists for operations, even for emergencies.

Wound coverage is delayed to allow for swelling and to facilitate a second-look procedure to reassess tissue viability. Using standard protocols, grade 3B/C open tibial fractures often require 30 to 50 weeks for consolidation^12^ with a 46% incidence of the delayed union if accompanied by the neurovascular deficit, as opposed to 16% if the vessels are intact^13^. Rates of deep infection could range from 10% to 50% depending on the nature of the injury, degree of contamination, as well as the age of the patient and associated co-morbidities^14^. Although widely accepted, this treatment protocol has been recently challenged, as repeated debridement and delayed closure could lead to additional tissue loss from desiccation and infection^15-18^.

Orthopaedic, plastic and vascular intervention must go hand in hand to treat these complex injuries^15,16,18^. The orthopaedic management of these severe injuries is undergoing a progressive change from external to internal fixation with increasing experience. Primary plating or primary nailing is now being preferred in Grade-1 to Grade-3A fractures, and sometimes even in grade-3B injuries^19-21^, despite the significantly high infection rates and the high re-operation rates compared to external fixation^22-24^.

Primary interlocking nails remain confined to Grade 1 to Grade 3A open fractures without significant bone loss and simple fracture patterns. Some studies^11-27^ have also correlated reaming with thermal damage to the bone, increased infection rates and decreased union rates. The staged conversion from temporary external fixator to interlocking nail is being recommended for poly-trauma patients^25^. However, in isolated severe Grade 3B/C open tibial fractures in the developing countries, this staged surgery often becomes a logistic problem due to overcrowding of operating theatres. Additionally, questions regarding the best time to convert an external fixator to an interlocking nail remain unanswered^28^, with the literature highlighting deep infection rates of up to 17%^29^ due to residual pin tract infection.

External fixators are perhaps the most preferred initial treatment modality in Grade 3B/C open tibial fractures; these have sometimes been used as the primary definitive fixation^30^, with no significant difference in non-union and deep infection rates as compared to interlocking nails^24^. Modern plastic surgery has advanced from the complexities of the pedicled to micro-vascular techniques, including free tissue transfers^31^. This allows aggressive use of flaps to rapidly and reliably convert a severe open fracture to closed injury in a single intervention, even with an external fixator in situ.

Using these techniques, we employed a single-stage protocol of emergent radical debridement and primary stabilisation with locally sourced external fixator constructs, along with immediate wound coverage using various types of flaps and vascular surgery intervention, when required. This protocol was used to manage grade 3B/C open tibia fractures at our tertiary care centre by a multidisciplinary team of orthopaedic surgeons, plastic surgeons and vascular surgeons. Our experiences and outcomes over a five-year study period are presented.

## Materials and Methods

The study was performed at Advanced Trauma Center, PGIMER, Chandigarh, starting from January 2013 and spanning over five years, during which, 38 patients with isolated severe open tibial shaft fractures (Grade 3B and 3C) without systemic injury, presenting to our centre were enrolled in the initial two years of the study. All cases were followed up and assessed for functional outcomes at three years from enrolment. Diaphyseal and metaphyseal open tibial shaft fractures were included, and intra-articular fractures were excluded. Ethical clearance from the ethics committee of the institute for this study was obtained. Consent was taken from all the patients before enrolling for the study.

A multidisciplinary team consisting of orthopaedic, plastic and vascular surgeons collectively managed all patients. Preoperative wound toiletry, antibiotics and tetanus prophylaxis were administered to all patients. Penicillin was added if anaerobic contamination was suspected, especially in farmyard injuries. All patients were taken to the operation theatre within one hour of presentation to our hospital after the necessary investigations including an angiogram, if needed.

Meticulous radical wound debridement was performed, both inside and outside the zone of injury with liberal application of lavage as per standard protocols. As viability and vascularity of the soft tissues were of prime importance during debridement, all devitalised tissues were excised freely inside the zone of injury. Debridement was further extended outside the zone of injury until adequate bleeding and viable tissues were encountered, to provide a healthy bed for tissue transfer. Skeletal stabilisation was done using stainless steel external fixator constructs appropriate to the fracture morphology from INOR, India. After debridement, intra-operative wound cultures were sent, for targeted postoperative antibiotic administration with the results, in the wards. After achieving physiological length, alignment and rotation, fractures were stabilised with maximum possible cortical contact achievable intra-operatively without significant shortening. Immediate wound coverage was provided in the same sitting by plastic surgeons. The choice between a fasciocutaneous flap or muscle flap, either local or distant, was based on the injury and soft tissue status. Distant micro-vascular free muscle flaps were preferred due to the fear of vascular compromise and poor viability at the zone of injury. The urgent vascular repair was done in Grade 3C open fractures as a priority along with the initial fixation and wound coverage using the same protocol, in the same sitting.

Post-operative intravenous antibiotics were used in all patients for the initial three days, including the higher spectrum antibiotics of piperacillin- tazobactam, linezolid and clindamycin for highly contaminated wounds followed by oral antibiotics till sutures were removed. Subsequent antibiotics were given according to the results of the intra-operative microbiological cultures. An aggressive postoperative dressing regimen was followed which consisted of regular wound examination, minor local debridement when necessary, close look for flap viability, customised aggressive wound care with foam dressings for exudating wounds, hydrogels to remove slough and promote autolytic debridement, and silver dressings. Negative pressure wound therapy (NPWT) was used in consultation with plastic surgeons for the removal of exudates, minimising venous congestion of flaps and promoting granulation tissue. Repeat debridement was done only in cases with elevated leukocyte counts along with clinical signs of infection. Patients were discharged after suture removal and were followed weekly for two months, and after that at two-monthly intervals till they recovered.

Fixators were kept in-situ for a minimum of four months in patients with at least two or more cortices in contact at the time of initial stabilisation. The early movement of the knee and ankle joints were encouraged; axial dynamisation and loading were individualised. Toe-touch was encouraged as soon as possible post-operatively depending on the wound status, and partial weight-bearing was started at six weeks, going to full weight bearing by three months.

Final clinico-radiological outcomes were assessed at the three-year follow-up using the Johner and Wruhs criteria^32^ (Table I). The results were compiled at the end of the five-year study period and compared with those of conventional protocol available in the literature, as well as with previous studies based on a similar concept. Clinical criteria for union were the ability of the patient to bear weight on the injured limb and perform activities of daily living, with no pain at the fracture site on palpation and physical stress. Radiological bridging of at least three cortices on standard AP and lateral views, with partial obliteration of the fracture line, was taken as a reliable criterion for fracture healing. After confirmation of union, the external fixator rods alone were removed with the pins left in place, and the patients were then instructed to bear full weight. If there were no symptoms or pain, the pins were subsequently removed after four days. Patients with persistent pain at the fracture site and with no evidence of callus formation at six months follow-up were labelled as delayed or as non-union and were planned for a second stage surgery. Functional and social outcomes were further documented based on subjective limitation of the activities of daily living like household work, family and leisure activities along with self-care. They were graded as having no difficulty, having some difficulty, or having an inability to perform these activities.

**Table I:** Johner and Wruhs criteria for clinico-radiological outcomes

CRITERIA	EXCELLENT	GOOD	FAIR	POOR
Non-Union	None	None	None	Yes
Neuro-Vascular Deficit	None	Minimal	Moderate	Sever
**DEFORMITY**				
Varus Valgus	None	2-50	6-100	>100
Anterior/Posterior	0-50	6-100	11-200	>200
Shortening	0-5mm	6-10mm	11-20mm	>20mm
**FUNCTION**				
Knee	Full	>90%	90-75%	<75%
Ankle	Full	>75%	75-50%	<50%
Pain	None	Occasional	Moderate	Severe
Gait	Normal	Normal	Mild Limp	Significant Limp

## Results

Four patients (grade 3B) were lost to follow-up within a month of enrolment and one patient (grade 3C), with injury to the tibio-peroneal trunk presenting after five days of injury underwent primary amputation. The remaining 33 patients were managed by our standardised protocol of “Fix and Flap” and followed up for a minimum of 36 months; 15 were grade 3B (45.4%), and 18 were grade 3C (54.5%) open fractures. There were 30 men (91%) and three women (9%) with a mean age of 35.3 years (range 18-60 years). The mean time of presentation after injury to our centre was 15.8 hours, with three patients presenting after 48 hours. Extensive degloving was seen in 11 patients (33%). Details of the 33 patients with their outcomes are shown in Table II.

**Table II:** Details of the 33 patients with Grade 3B/C open tibial fractures

SNo.	Age	Open Grade	AO Type	Time Of Presentation	External Fixation#	Flap*	Surgery Time	Union	Time to Union	Secondary Procedure	Total Surgeries	Deep Infection	Outcomes
1	23	3b	42b3	3h	U	ALT	8h	U	38wk		1		EXCELLENT
2	42	3b	42b3	8h	U	Hemisoleus	6h	U	16wk		1		GOOD
3	31	3c	41a2	2h	H	Gastrocmenius	9h	u	72wk	PLATING+BG	3		FAIR
4	21	3c	42c2	7h	H	LD	8h	u	42wk		1		GOOD
5	33	3b	43a3	48h	H	ALT	11 h	u	32wk		1		EXCELLENT
6	57	3b	42a3	9h	U	ALT	12h	u	44wk	ILN+BG	2		POOR
7	36	3c	42a2	1 h	U	ALT	17h	u	15wk		1		FAIR
8	42	3b	42c2	7h	u	LD	12h	u	18wk		1		GOOD
9	25	3b	42c3	8h	H	ALT	9h	u	76wk		2	Yes	POOR
10	58	3c	41a2	10h	T	Gastrocnemius	8h	u	35wk	PLATING+BG	2		EXCELLENT
11	59	3c	42b3	3h	U	ALT	13h	u	70wk	PLATING+BG	5		POOR
12	43	3c	41a3	9h	T	Gastrocnemius	7h	u	33wk		1		EXCELLENT
13	44	3b	42c2	36h	H	ALT	15h	u	22wk	ILN+BG	2		FAIR
14	21	3c	43a3	18h	H	ALT	11 h	u	68wk	ILIZAROV+BT	4		POOR
15	36	3b	42b2	38h	U	LD	9h	u	19wk	ILN+BG	2		EXCELLENT
16	19	3c	42c2	6h	U	GRA	12h	u	16wk		1		GOOD
17	60	3b	43a2	38h	T	RADIAL	10h	u	78wk		5	Yes	POOR
18	32	3b	43a1	18h	T	ALT	13h	u	21wk		1		GOOD
19	29	3c	42b2	3h	U	LD	11 h	u	56wk	ILIZAROV+BT	4		POOR
20	47	3c	42c3	5h	H	ALT	16h	u	23wk		1		EXCELLENT
21	50	3b	41a3	48h	H	CL	8h	u	42wk	PLATING+BG	3		FAIR
22	36	3c	42c3	10h	U	ALT	10h	u	48wk		1		GOOD
23	41	3b	41a2	72h	T	ALT	13h	u	44wk	PLATING+BG	2		GOOD
24	47	3c	42b3	11 h	U	GRA	11 h	u	62wk	ILIZAROV+BT	3		POOR
25	39	3b	43a2	12h	T	RSA	6h	u	17wk		1		EXCELLENT
26	22	3c	42b2	1 h	U	ALT	14h	u	22wk	ILN+BG	2		EXCELLENT
27	19	3b	42b1	8h	U	ALT	11 h	u	88wk	ILIZAROV+BT	2	Yes	POOR
28	37	3c	41a3	14h	T	RADIAL	8h	u	18wk		1		FAIR
29	18	3c	42c3	3h	H	FC	9h	u	61wk	ILIZAROV+BT	2		EXCELLENT
30	22	3c	43a3	9h	H	FC	7h	u	16wk		1		GOOD
31	38	3c	43a1	8h	T	ALT	13h	u	19wk		1		FAIR
32	21	3b	42c2	42h	H	LD	11 h	u	69wk	ILIZAROV+BT	2	Yes	GOOD
33	18	3c	42b3	7h	U	CL	16h	u	30wk	ILN+BG	2		EXCELLENT

*FLAPS- GRA-Gracilis, LD-Lattisimus Dorsi, ALT-Anterolateral Thigh, CL-Cross-Leg, RSA-Reverse Sural Artery, FC-Fasciocutaneous

#EXTERNAL FIXATORS- U-Uniplanar, H-Hybrid, T-T-Type Biplanar, BG-Bone Grafting, BT-Bone Transport, U-United

**Fig. 1:**
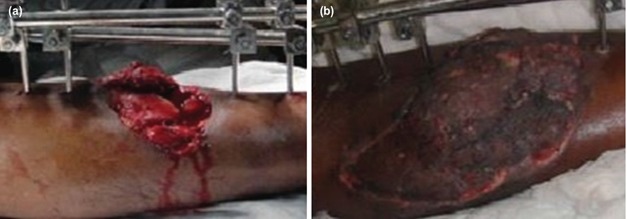
(a,b) Grade 3B open tibia fracture managed by external fixation and distant free muscle flap (Gracilis) in the same sitting.

**Fig. 2:**
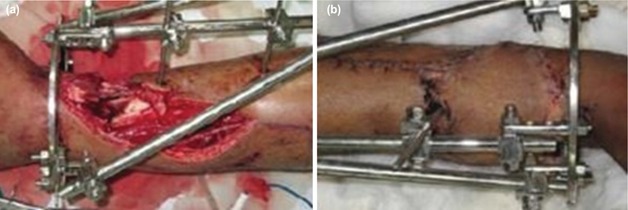
(a,b) Grade 3B (43A3 – Complex Distal Extra-articular) open tibia fracture managed by external fixation and distant free muscle flap (Anterolateral Thigh) in the same sitting.

**Fig. 3:**
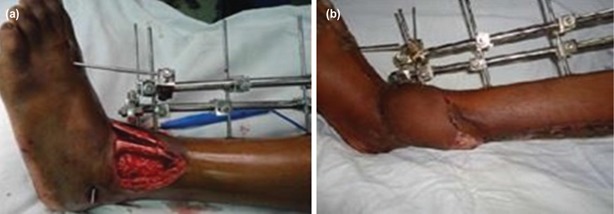
(a,b) Distal 1/3rd Grade 3B (43A1) open tibia fracture managed by external fixation and fasciocutaneous flap (Reverse sural artery based).

Immediate soft-tissue cover was achieved in 24 patients with distant free muscle flaps, 15 anterolateral thighs, five latissimus dorsi, two gracilis and two radial artery based forearm flaps; in four patients with local rotational muscle flaps, three gastrocnemius, one hemi-soleus; and in five patients with fasciocutaneous flaps, two perforator based, two cross-leg and one reverse sural artery based (Fig. 1-3). The average duration of surgery was 10.7 hours. Seven patients who presented later than 24 hours of injury were also provided with an immediate flap cover at the time of initial surgery.

**Fig. 4:**
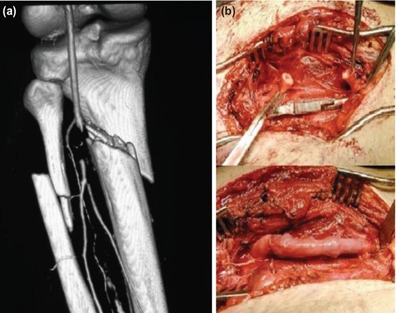
(a) CT Angiogram showing Injury to tibio-peroneal trunk in Grade 3C open fracture (b) managed by thrombectomy followed by reverse saphenous vein graft.

**Fig. 5:**
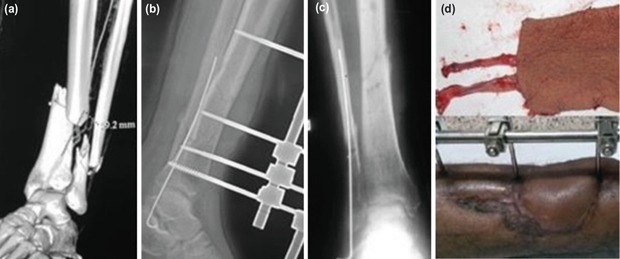
(a,b,c,d) Grade 3C open tibia fracture with ATA injury managed by external fixation, urgent vascular repair and radial artery based forearm flap. Union was achieved with primary definitive external fixation with a single major surgical procedure.

Eighteen patients had an associated arterial injury, six with tibio-peroneal trunk, eight with posterior tibial artery and four with anterior tibial artery, which was repaired urgently after an initial fracture stabilisation, before proceeding for the wound coverage. Vascular surgeons carried out all these repairs below the level of popliteal trifurcation (Fig. 4 and 5).

Bony stabilisation was achieved using simple uniplanar external fixator constructs in 14 patients, and hybrid multi-planar construct with ring and tubular rods in 11 patients and T-Type bi-planar construct with convergent pins in 8 patients. Early fixator removal along with secondary stabilisation procedure was done in 8 patients who had an initial joint spanning fixation with bone loss after soft tissue healing was obtained.

**Fig. 6:**
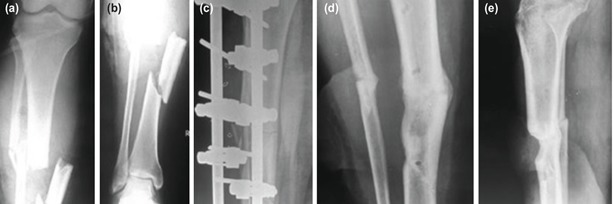
(a,b,c,d,e) Grade 3C open tibia fracture with injury to PTA managed by emergent debridement, skeletal fixation followed by urgent vascular repair and LD free flap for wound coverage. Union was achieved with primary definitive external fixation in 42 weeks.

**Fig. 7:**
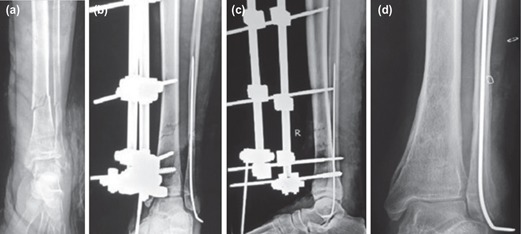
(a,b,c,d) Grade 3B open tibia fracture united at 23 weeks with primary definitive external fixation and immediate wound cover with RSA flap.

**Fig. 8:**
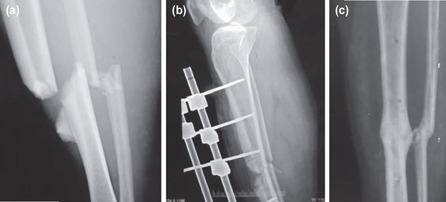
(a,b,c) Union in Grade 3C open tibia fracture at 33 weeks with primary definitive external fixator and immediate flap coverage.

Limb salvage and union was achieved in all 33 patients at the end of the five-year study period. Average time to union was 40.3 weeks (16-88), which was comparable to the literature-based results of the standard protocols (30-50 weeks)^12, 13^, with no statistically significant difference noted. Fifteen patients (45.4%) united with only a single major surgical procedure utilising primary external fixators as the definitive fixation (Fig. 6, 7 and 8).

**Fig. 9:**
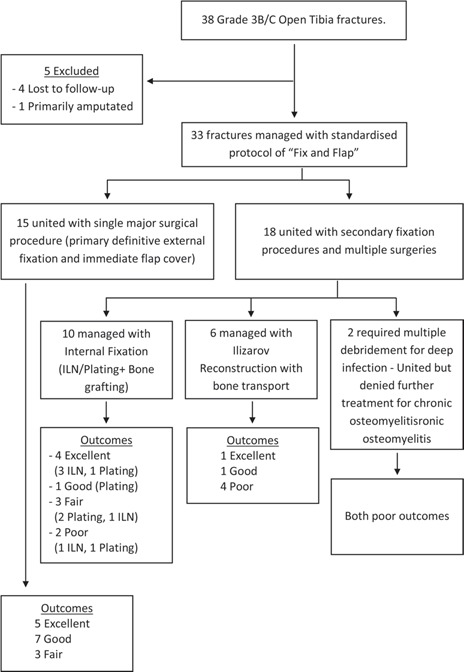
Flow chart showing outcomes of various surgical procedures.

Flap failure was seen in three patients (9%) within a week of surgery, necessitating revision coverage with cross-leg flap as a salvage procedure; the other 30 flaps settled over in due course of time. Superficial infection was seen in eight patients (24.2%), which resolved with the aggressive postoperative dressing regimen with the microbiological culture-based antibiotic usage, foam/hydrogel/silver dressings and negative pressure wound therapy (NPWT) in consultation with the plastic surgeons. Pin tract infection was seen in two patients (6%) who had to undergo antibiotic infiltration and minor debridement. Deep infection was seen in four patients (12.1%) with Staphylococcus aureus isolated in two, and klebsiella and pseudomonas, in one patient each. Of these four patients, two with a united fracture but chronic discharging sinus refused any further intervention; the remaining two (6%) were managed by debridement and sequestrectomy, followed by Ilizarov reconstruction with bone transport for a final union. These two patients had grossly contaminated wounds with extensive degloving at the time of initial presentation. Both superficial and deep infection rates in our study were similar to the results of the standard published protocols^14,33^ without any statistically significant difference. Sixteen patients (48.4%) underwent secondary stabilisation procedures with plating, ILN, or Ilizarov reconstruction. Eight of these patients had a neurovascular deficit at initial presentation. Bone grafting with a fibula or iliac crest was required in ten patients. Outcomes of various surgical procedures are shown in Fig. 9.

Malunion with varus deformity (>10^o^) was seen in two patients and pro-curvatum deformity (>20^o^) in one patient. However, malunion rates (9%) in our study were significantly lower than those reported in the literature using standard protocols (33.3%)^24, 33^. Shortening >2.5cm was seen in three patients with comminuted fractures and severe bone loss from the initial injury.

Using the Johner and Wruhs criteria, 19 patients had excellent to functional outcomes (57.5%), six patients had fair outcomes (18.1%), and eight patients had poor outcomes (24.2%) (Table III). A total of 23 patients (69.6%) faced occasional difficulties in carrying out the activities of daily living of household work, family or leisure activities. None of the patients was bedridden or had severe pain at the fracture site.

**Table III:** Details of functional outcomes of our study as per Johner and Wruhs Criteria

**A. Excellent to Good (19/33)** Normal gait No/occasional Pain Able to perform all activities of daily living Full/mild restriction in knee (>90%) and ankle (>75%) ROM **Fair (6/33)** Mild Limp Moderate Pain Limitation in Strenuous activity Restricted knee (75-90%) and ankle (50-75%) ROM **Poor (8/33)** Multiple Surgeries Deep infection (3/8) Shortening >2.5 cm (3/8) Restricted knee (<75%) and ankle (<50%) ROM Significant limp with pain

## Discussion

Severe open tibial shaft fractures are a major management challenge, especially in developing countries with limited access to tertiary care facilities. A major advance in management came in 1973 with the introduction of microvascular free flaps by Daniel and Taylor^34^. Godina^31^ brought a new dimension to the treatment of these injuries by advocating early free tissue transfer within five days of trauma. This reduced the time to union and the incidence of infection^35, 36, 37^. It should be emphasised that timing of early soft tissue reconstruction is still debatable with some studies advocating coverage within three to five days of injury^29,34,37,38,39^ and others favouring immediate wound cover at the time of initial surgery^21,40^. Our five-year study supported the observation that if the patient were hemodynamically stable, delay in soft tissue cover was unnecessary, as it could lead to additional soft tissue loss and a further increase in chances of wound contamination. Hence, there should be aggressive surgical management to tackle these complex injuries without delay^41^.

As local flaps have four times higher risk of wound complications than free flaps^42^, we preferred distant free muscle flaps (24) in the majority of our patients followed by fasciocutaneous (5) and local rotational muscle flaps (4) for immediate wound cover. Our study showed that this aggressive management of the severe open tibial shaft fractures was an effective modality, with favourable outcomes in the majority of patients. We accept that this approach was radical and that immediate wound coverage along with debridement and initial fixation in the same sitting had many potential complications^2,35,36,38,42,43,44^. However, due to financial as well as logistic issues, including long operation wait-lists endemic in underdeveloped countries, this single-stage approach would be better suited for overcrowded tertiary care centres in developing countries. Recent studies^45-47^ also emphasised the importance of single-stage definitive ortho-plastic reconstruction in severe open tibial fractures leading to good outcomes and significantly decreased infection rates.

Our patients presented at a mean time of 15.8 hours from injury to our centre and were subsequently taken to the operation theatre within an hour of arrival as a priority. All cases were managed with external fixation, wound coverage and vascular intervention, if needed, at the time of the initial surgical intervention using a single-stage standardised protocol by a multidisciplinary team of orthopaedic, plastic and vascular surgeons. Comparing the results of our five-year study with conventional standard protocols advocating serial debridement and delayed wound cover^11, 12, 14, 48^ our mean time to union and infection rates were comparable. Additionally, there was no need for multiple serial debridements and delayed fixation surgeries in many cases, as 15 patients (45.4%) underwent only a single definitive surgical procedure. Hence, the option of a single-stage surgery became fairly attractive in terms of patient care and hospital logistics.

Hertel *et al*^17^ presented a smaller comparative study with a mean follow-up of 47 months comparing immediate versus delayed fixation of open tibial fractures. They encountered increased mean rates of infection (4 versus none) as well as the increased mean rate of secondary surgical procedures (3.9 versus 1.6) in the cases where fixation was delayed using standard protocols, compared with those operated with immediate fixation and wound coverage. Gopal *et al*^49^ presented a retrospective study assessing functional outcomes of grade 3B/C open tibia fractures with a mean follow-up of 46 months, advocating early wound coverage along with primary internal fixation. Forty per cent of their cases underwent a single definitive surgical procedure of emergent internal fixation and flap coverage. Their infection rates (6.1% vs 12.1%) and functional outcomes of joint stiffness, union rates and pain) were comparable to our study. A slightly higher initial flap failure rate requiring revision flap surgery in our study (9% vs 3.5%) could be due to gross contamination combined with a delayed presentation in many of our cases. Nevertheless, all flaps settled over in due course of time. Toia *et al*^50^ retrospectively compared the results of combined orthoplastic versus staged protocol and concluded that external fixation and free flaps could be successfully integrated to give better outcomes as compared to staged protocol. Their average time to union was ten months as was in our study, and infection rates of 17% and 12.1% were comparable in both the studies.

Primary definitive external fixation had been used successfully as a treatment modality in the literature30,51 with good union rates and fewer complications comparable to our study; however, a combined protocol of best choice of fixation and timing of wound coverage in these severe injuries was still missing in the literature. It was important to note that we preferred the low cost but the equally effective primary modality of stable external fixation in all our cases, which provided similar long term results, especially when done as a primary definitive surgical procedure in the 15 patients (45.4%). Six of these patients also underwent vascular repair for Grade 3C injury. Excellent to good outcomes were seen in the majority of our patients (57.5%). We also encountered problems associated with external fixators like pin tract infection (6%) and mal-union (9%). These warranted minor surgeries, but overall had no impact on fracture healing or deep wound infections.

Factors associated with increased risk of infections and other complications following open tibial fractures included Grade 3B/C injuries, gross contamination and systemic comorbidities^52^. Prevention of infection and early fracture healing depended on the adequacy of the debridement, targeted antibiotic usage, stable skeletal fixation^53^ and immediate obliteration of the dead space by a healthy soft tissue cover. Keeping these principles in mind, a team of surgeons using their clinical skills, at the first stage itself as part of a clear, standardised management protocol, played a major role in guiding these severe injuries on the road to favourable outcomes.

The relevance of this study arose from the fact that a long-term prospective study from the developing countries emphasised that outcomes of severe Grade 3B/C open fractures managed with a single-stage standardised “fix and flap” protocol using external fixators and immediate wound coverage was missing in the literature. Most of our problems were associated with a delayed presentation with a mean time of 15.8 hours of patients, extensive degloving of soft tissues (33%) and associated neurovascular deficit (54.5%) and gross contamination. Despite these factors, this single-stage management protocol for complex fracture conventionally treated by multiple surgeries would be a good option at overcrowded tertiary care centres with long waiting lists for surgery.

## Conclusion

The use of single-stage radical protocol of emergent “Fix and Flap” using external fixation and immediate wound coverage as primary modality, gives good results in complicated scenarios of delayed presentation, gross contamination, neurovascular deficit and extensive degloving. This single-stage protocol gave excellent to a good outcome in the majority of patients and may be a better management strategy in the overcrowded tertiary care centres of developing countries with a single major intervention.
